# Personalized medicine and hospitalization for heart failure: if we understand it, we may be successful in treating it

**DOI:** 10.1002/ejhf.1463

**Published:** 2019-04-11

**Authors:** Faheem A. Ahmad, Mark C. Petrie, John J.V. McMurray, Ninian N. Lang

**Affiliations:** ^1^ BHF Glasgow Cardiovascular Research Centre, Institute of Cardiovascular and Medical Sciences University of Glasgow Glasgow UK

Randomized trials in patients with hospitalized heart failure (HHF) continue to frustrate the cardiology community. Promising haemodynamic, structural and biomarker findings from phase 2 studies consistently fail to deliver substantive benefits in larger outcome trials. What underlies these recurrent failures? Why are persistently high readmission and post‐discharge mortality rates not being reduced? Challenging any pre‐conceived ideas about the existence of a ‘typical’ acutely decompensated heart failure patient is fundamental, as is the adoption of a carefully personalized approach. These individuals come from a remarkably heterogeneous group. Surely precise phenotyping should translate to a more successful therapeutic approach?

## Lack of robust consideration of the presenting phenotype

**Figure 1:**
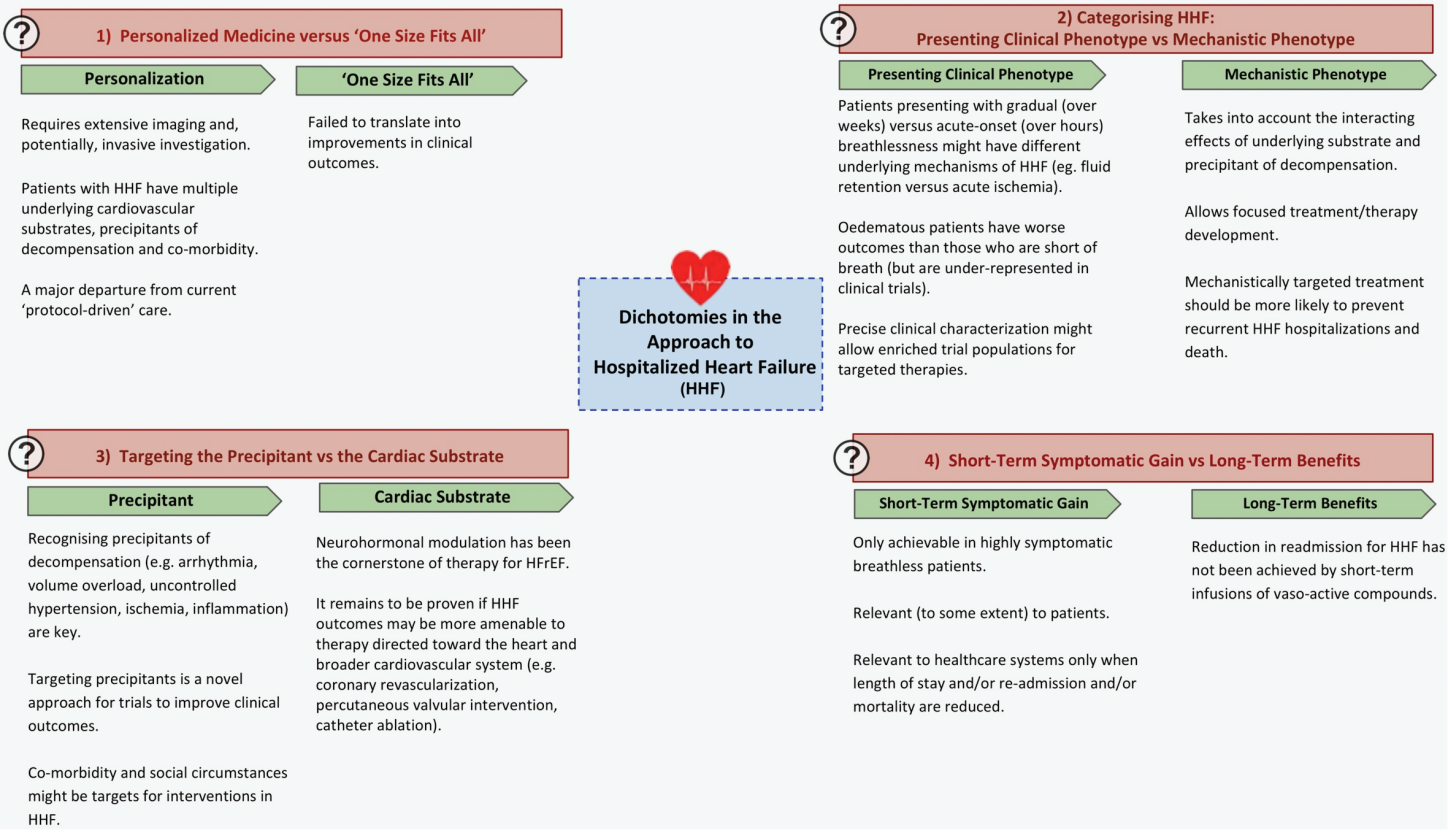
Dichotomies in the approach to hospitalized heart failure (HHF). HFrEF, heart failure with reduced ejection fraction.

In our opinion, it is unrealistic to expect a change in outcomes until a personalized approach is adopted (*Figure* 1). This includes categorizing the heart failure aetiology, presenting clinical phenotype and precipitant of hospitalization. Detailed and precise investigation of the heart, broader cardiovascular system and co‐morbidity is required (*Table* 1). Once the reason for HHF is known, existing treatments can be applied (primarily loop diuretics and nitrates) and novel investigational drugs and techniques or devices can be assessed. Applying one therapy in patients with different aetiologies and mechanisms has been surprisingly successful in the more homogeneous ambulatory population with chronic heart failure and reduced ejection fraction (HFrEF), but in HHF this broad‐brush approach has not been effective.

**Table 1:** Personalized assessment of hospitalized heart failure

**System**		**Assessments**
Cardiac	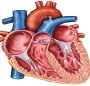	Left and right ventricular and atrial function Rhythm QRS durationCoronary vasculature Epicardial and microvascular Valvular assessment Intra‐cardiac pressures Pericardial assessments
Vascular	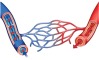	Arterial blood pressure Pulmonary pressures and haemodynamics Venous pressures Cardiac preload, renal afterload Peripheral vascular disease Aorto‐iliac, mesenteric Renovascular disease Cerebrovascular disease
Co‐morbidity	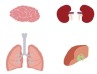	Renal function Hepatic structure and function Diabetes Lung disease Anaemia/iron deficiency Obesity Infection Cancer
Psychological	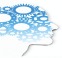	Psychological (anxiety, depression) Cognitive impairment Frailty Adherence to medication
Social	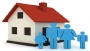	Housing Isolation Access to healthcare services

Patients who present primarily with dyspnoea at rest comprise up to 42% of ‘real‐world’ patients with HHF in registries^1^ but over 90% in randomized controlled trials.^2^ Those who present with peripheral oedema as their main complaint and who are only breathless on exertion have worse outcomes than those with pulmonary congestion and consequent breathlessness at rest.^1^ In clinical trials, it is likely that the under‐representation of patients with predominant peripheral oedema dilutes treatment effects. We would argue that there is a growing need to separate these groups in the design of trials assessing mechanistically‐targeted therapies.

Differentiation of those with a slower ‘sub‐acute’ decline in their condition from those with ‘acute’ breathlessness has only recently received attention.^1^ Perhaps those who present with increasing symptoms over weeks or months have fluid overload as their primary therapeutic target. The minority who present with a true ‘acute decompensation’ in the absence of a slow, often sub‐clinical, accumulation of fluid might have uncontrolled hypertension, unstable coronary disease or an acute arrhythmia as a precipitant, and thus require quite different treatment approaches. It seems logical that targeting the cause or causes of decompensation should yield better long‐term benefits than the undifferentiated treatment of the resulting signs and symptoms.

HFrEF and heart failure and preserved ejection fraction (HFpEF) represent different cardiac phenotypes that are likely to respond to distinct therapeutic interventions. However, providers may be criticized for failing to think ‘beyond the ejection fraction’. For example, a patient with HFrEF as a result of a large prior myocardial infarction and scar could respond differently to a therapy than might a patient with HFrEF due to an acute toxic insult, myocarditis, or poorly controlled systemic hypertension. Establishing the incremental benefits of such phenotypically‐targeted therapies (in addition to renin–angiotensin–aldosterone system inhibitors and beta‐blockers in HFrEF) would require stricter trial inclusion criteria.

## Over‐optimistic expectations of the long‐term benefits of short‐term infusions

Many clinical trials in HHF have investigated short‐term infusions of vasoactive therapies^3^ with attractive biological profiles. These have been drugs that may well have produced short‐term effects on the heart, vasculature and kidneys. In retrospect, it was perhaps overly optimistic to think that a short‐term infusion might result in a reduction in major clinical events such as death or recurrent HHF. However, short‐term therapies can reduce inpatient mortality and morbidity, which is sustained in the longer term in patients with acute myocardial infarction.^4^ It is, therefore, not impossible that short‐term treatment can have long‐term benefit, but demonstration of the small effects of beta‐blockers^4^ and angiotensin‐converting enzyme inhibitors^5^ in acute myocardial infarction required the randomization of large numbers of patients. Alternatively, continuation of oral therapy after early intravenous infusion might be beneficial in HHF (with beta‐blockers in acute myocardial infarction another analogous example).^6^

## Possible cardiac targets in hospitalized heart failure

Exciting times may lie ahead. Careful phenotyping (*Table 1*) could lead to trials of new therapies in tightly defined groups. Studies of catheter ablation for atrial fibrillation could be launched in both HHF secondary to HFrEF, HFpEF, or both. Trials of coronary revascularization with percutaneous intervention or coronary artery bypass grafting are overdue and could be performed in HHF – again in HFrEF, HFpEF, or both. Impressive benefits of transcatheter mitral valve intervention have been demonstrated in very carefully selected patients with chronic HFrEF.^7^ It remains to be established whether these benefits can be extended to the HHF population, and the potential beneficial effects of tricuspid and aortic valve interventions remain undefined in this group.

## Possible co‐morbidity targets in hospitalized heart failure

Many co‐morbidities have long been recognized to be targets for treatment in HHF including diabetes, lung disease, renal failure, iron deficiency and anaemia, among others. Indeed, trials investigating the potential benefit of sodium–glucose co‐transporter 2 (SGLT2) inhibition in diabetic patients with HHF^8^ and of intravenous iron in HHF are ongoing.^9^ The role of lung infections in precipitating HHF is well recognized.^10^ However, the protective effects of influenza vaccination upon HHF are surprisingly poorly defined. This is the focus of an ongoing trial including patients with heart failure and comparing the effect of high vs. low‐dose influenza vaccination upon all‐cause mortality and cardiopulmonary hospitalization.^11^

Many of the co‐morbidities found in patients with HHF have not been perceived to fall within the purview of cardiology but recognizing them may improve outcomes. Perhaps early diagnosis of cognitive impairment or psychological conditions could provide benefit. Establishing the social environment of a patient can reveal obvious ways to influence readmission.^12^ Can the patient afford the drugs they are prescribed? Do they have ready access to a pharmacy? How deep is their social support network?

## Research to target poor outcomes after discharge from hospital

Outcomes remain poor after discharge from an admission with heart failure.^13^ This high‐risk period is an opportunity for more research to understand causes of rehospitalization and death. Future treatments may potentially be targeted at these events. What proportion of deaths and readmissions are due to rhythm disturbance? We need to better understand what hospitalization represents – is it just a marker of a patient at higher risk or does the deterioration leading to admission (or what happens in hospital, or both) directly depress the life trajectory of these patients? Does irreversible cardiac, renal or other organ or tissue damage occur? Can this risk be modified? Perhaps earlier implantation of a defibrillator or cardiac resynchronization therapy device yields benefit in select cases? How many patients have persistent fluid overload at discharge and might benefit from more aggressive diuresis (perhaps from SGLT2 inhibitors rather than bigger doses of loop diuretics)? Persistent unrecognized fluid overload could be identified by lung ultrasound.^14^ In patients with early and frequent readmission, wireless pulmonary artery pressure monitoring, or other techniques might be assessed as a prevention strategy.

A key focus should be to improve equity of access to heart failure management programmes, including follow‐up by a heart failure nursing team and allied health practitioners. By taking this more comprehensive approach, the psychological, social and economic issues that might negatively influence outcomes should become clearer. Research to address many of these questions is overdue and the bulk of currently available evidence is based on subgroup analysis which is, therefore, hypothesis‐generating and not definitive.

## Expanding the use of mechanical circulatory support in hospitalized heart failure beyond their present niche

Durable ventricular assist devices (VADs) remain a niche therapy for few and highly selected patients in a small number of centres in the United States, Germany and some other countries. Notwithstanding the considerable cost of these devices and substantial resources required to care for patients post‐implant, there is a major opportunity to test durable VADs against conventional HHF treatment in patients towards the sickest end of the spectrum. The mainstay of therapy for these patients is inotropes – some of which are known to increase rather than decrease mortality. A trial of durable VADs vs. inotropes in countries where VADs are not used or funded would potentially lead to convincing evidence of the benefit of these devices as destination therapy (to date only 129 patients have been randomized to durable VADs or medical therapy).^15^ Intra‐aortic balloon pumps may also have a role in HHF but no trial has yet been performed (a trial of cardiogenic shock in acute myocardial infarction – a very different condition – was neutral).^16^

## Conclusion

Trials in HHF have resulted in failure. The ‘typical’ HHF patient does not exist. A change of direction is necessary with a new era of personalized assessment. Management of these complex patients requires assessment that is not limited to the presenting features but extended to the nuances of aetiology, precipitants of decompensation and co‐morbidity. Only by doing so will we identify phenotypically similar groups of patients with HHF who can enter trials to understand which pharmacological, monitoring or device therapies might finally lead them to a brighter prognosis.

**Conflict of interest:** none declared.
